# Medical Opinion and Movement

**Published:** 1910-02-05

**Authors:** 


### Medical Opinion and Movement.

DR. BERGRATH, of Breslau, advises the Administration of Quinine Hydrochloride in cases of Pemphigus. Four severe cases of the disease are reported from the dermatological clinic at Breslau, in which the usual drugs, including arsenic preparations, failed to relieve the condition; but in each case the disease yielded to quinine hydrochloride given in doses of 4 to 8 grains three or four times a day. In one case the disease took the so-called malignant form, and was generalised all over the body, involving the mucous membranes as well as the skin. Under the influence of the quinine the patient began to improve on the third day. Fresh bullæ continued to appear for a few days, but of lesser dimensions, and the older ones commenced to dry up and disappear. The dose was increased gradually to 8 grains four times a day, and at the end of a fortnight the patient was quite convalescent. In this case and in a second case there was a rise of temperature at the beginning of the treatment, but this quickly subsided. In three of the cases the quinine was well borne without any of the usual signs of intolerance; but in the fourth case after treatment for a few days the quinine had to be suspended on account of buzzing in the ears and general lassitude. Three days later the treatment was again attempted, but with the same result. As, however, the disease assumed then a very severe form on cessation of the treatment, and the general condition of the patient became serious, the treatment with quinine was tried again for the third time; and this time, curiously, it was quite well tolerated to the extent of 24 grains of quinine hydrochloride per day, and the patient made a good recovery.

---

THE Treatment of Pernicious Anæmia with Glycerine has been conceived on certain theoretical considerations, and apparently carried out with success by Dr. H. J. Vetlesen, of Christiania, at least in one case. No very powerful therapeutic properties have been hitherto attributed to glycerine. It is not the kind of medicament that might be expected to prove efficacious in a disease of such severity and obstinacy. The researches of Tallqvist and Faust appear to have suggested the idea to Dr. Vetlesen. According to them the destruction of the erythrocytes associated with pernicious anæmia is due to the hæmolysing action of a lipoid substance set free, or produced, by the causal agent of the disease. On the supposition that this lipoid substance would be oleic acid, it occurred to Dr. Vetlesen that glycerine should form a suitable remedy, as it would combine with the oleic acid to form a harmless ether-trioline. The case in which he tried the treatment was a woman aged 33. On admission, six weeks after confinement, she complained of lassitude, palpitations, vertigo, and troubles of vision. Temperature was raised, pulse 120. There was a systolic anæmia bruit, retinal hemorrhages, no œdema or albuminuria. Blood examination gave 990,000 erythrocytes and 2,000 leucocytes. There was poikilocytosis, numerous polychromatophile erythrocytes, and also granular basophiles. The hæmoglobin content was 20 per cent. The patient was given three tablespoonfuls of glycerine a day, with half the amount of lemon juice. In three weeks the hæmoglobin content was 60 per cent. and the red corpuscles 2,860,000. Thirty-nine days later the hæmoglobin amounted to 90 per cent. and the red corpuscles 4,760,000. The body weight increased from 46.5 kilos. to 58.2 kilos. The cardiac bruit disappeared, and the patient was quite well. More recently the attention of Dr. Vetlesen has been drawn to the fact that lemon juice has been recommended for pernicious anæmia as in scurvy. In subsequent cases, therefore, he will dispense with this addition, but in the meantime this case deserves to be placed on record.

---

DR. K. TASKINEN advocates Rubbing with Snow as a method of Treatment for Neuralgia, muscular and articular rheumatism and allied conditions. The affected part is first rubbed with the snow in the hand for two or three minutes and then quickly dried. After this the usual massage and gymnastic exercises may be carried out. The author has treated in this way 42 cases, including cases of sciatica, lumbago, and other forms of neuralgia and rheumatism. He has effected 32 cures, with improvement in six cases and four failures. He is convinced that the rapidity of cure was much more marked with this treatment than with ordinary massage. The most striking effect is upon the pains, which rapidly subside under the treatment. He explains this by the reaction produced by the rubbing. The cold causes a transitory anæmia by contraction of the cutaneous blood-vessels, which is followed by an active hyperæmia, and he thinks that this is more efficacious than the hyperæmia produced by the hot-air douche. When snow is not obtainable, which is certainly the rule in this country, ice may be used; but the author does not find it so efficacious as snow.

---

IN cases of Infantile Pyloric Stenosis rectal injections of saline solution have been advocated and successfully employed. Dr. Rosenstern, of the Children's Hospital at Berlin, prefers, however, to administer the saline solution by means of gradual instillation into the rectum, similarly to the method now generally adopted after abdominal operations. For this purpose an irrigator with a long rubber tube is used, provided with a stopcock and a long glass rectal tube. The rectal tube is passed up as far as possible and fixed around the anus by means of adhesive plaster, so as to prevent the fluid from escaping. The stopcock is turned on so as to allow the fluid to pass in drop by drop (30 to 40 drops a minute). The flow should be allowed to continue for about two hours twice a day. In this way about 500 c.c. ($\frac{3}{4}$ to 1 pint) of fluid can be introduced into the system. In addition to the sodium chloride the author also recommends potassium and calcium chloride. The composition of his saline solution is as follows: Sodium chloride 7.5 grammes, potassium chloride 0.42 grammes, and calcium chloride 0.24 grammes per litre of water. The object of this treatment in Dr. Rosenstern's mind was to combat the loss of water to the organism, but he found that from the commencement of the treatment these rectal instillations of fluid had a most beneficial effect upon the course of the affection. Vomiting quickly diminished, and at the end of three days disappeared altogether. In three subsequent cases the same good results followed from the treatment. Vomiting ceased and there was a marked improvement in the general condition.

Caries of the Hyoid Bone, and, indeed, any other lesion of it, must be an extremely rare event. Such a case has recently been reported by Dr. Cannaday, of Charleston, Virginia. A healthy young woman presented herself for the treatment of a sinus which had been present ever since the age of four years. There was no history of accident or injury. At operation the whole sinus, with an elliptical piece of the skin about its orifice, was dissected right out without opening it. This involved the removal of all the centre of the hyoid bone, which was detached from its posterior periosteum: the ends of each cornu were left. Recovery was uneventful, and it is of interest to note that there was no impairment of any muscular action; so that the muscles attached to the hyoid bone were able to make use of the two cornua and the periosteum of the body of the bone. Examination of the specimen revealed no evidence of any tuberculous process, but only evidences of chronic inflammation; the bone was decidedly carious. In another recorded case of caries of the hyoid bone, reported by Ullman, a similar piece of the bone was resected, and here also muscular power remained unimpaired. The same has been observed after the separation of sequestra, which occasionally follow acute suppuration in this region. New growths of the hyoid are so rare that only five or six have been recorded. Fracture is mentioned in textbooks, and is apparently very dangerous; but any gross lesion is sufficiently rare to be worth placing upon record.

The customary Preoperative Purge is strongly condemned by Dr. Walker, who says it is unnecessary in most cases, and should be given only when there is a clear indication for it. The practice is, he says, unnecessary and injurious; patients are made uncomfortable and are weakened. In addition to this, germ activity in the intestinal canal is stimulated just as in enteritis, so that if the intestine is opened during the operation the probability of infection from it is increased. There is also, according to this author, more post-operative tympany after purgation. A diet of digestible food for the twenty-four hours preceding operation, and a fast for the last eight to twelve hours, are what Dr. Walker recommends, except when there is an obstructive lesion of the intestine. In cases of milder fæcal stasis, short of obstruction, he would allow a purge or laxative a few days before operation, followed by enemas. But the routine use of any powerful drug is to be deplored, and the habitual preoperative purge is indefensible. The author's objections to purgation apply apparently to medical cases as well as surgical, and he is convinced that much harm is done in general practice by the prescription of purges; he fails to consider how constipation is otherwise to be combated, and seems to regard it as a condition which may be left to itself, an opinion which most practitioners will decline to endorse.

Some Notes on Post-Partum Hæmorrhage, contributed to the *American Journal of Obstetrics and Gynæcology* by Dr. Douglas Stewart, contain one or two novel suggestions as well as better known ones. He has observed uterine contraction set up by the old plan of putting the infant to the breast; and the use of two cupping glasses is also, he says, useful in the same way as a preventive measure. He warns against the adoption of this device at the same time as Credé's method is applied to the uterus, for the placenta may be shot out with such violence as to leave behind part of the membranes. Credé's method is often overdone, and does not demand the exercise of any great amount of pressure or force at all. An ounce of vinegar given by the mouth he has found valuable as a means of causing a prompt and firm contraction of the uterus. If the patient is unable to swallow he recommends that the fundus of the uterus be lifted forward against the anterior abdominal wall, the intestines pushed out of the way, and a hypodermic syringeful of vinegar injected straight into the uterine muscles through the linea alba of the abdominal wall. This drastic remedy is described as quite easy and free from bad effects; aseptic precautions are, of course, to be observed. Uterine action should begin in ninety seconds, and should be progressive. As soon as the patient can swallow, vinegar should be given by the mouth, and the uterine contraction then becomes tonic. Another device useful in emergency is to soak a sterile swab in chloroform, squeeze it nearly dry on a volsellum, and pass it into the uterus right up to the fundus. It must be withdrawn rapidly, because uterine contraction begins so quickly that otherwise there may be difficulty in getting it away. Autotransfusion by bandaging the extremities also comes in for a word of praise.

A Material which is attracting some notice just now as an aid to the microscopical search for Tubercle Bacilli is Antiformin. Uhlenhuth observed that this substance, a mixture of soda lye and javelle water, dissolves quickly and almost completely the very various tissue débris with which tubercle bacilli are usually associated, leaving the bacilli themselves intact, and with their tinctorial characters unaltered. Its effect depends upon powerful oxydation, and a useful strength to use is a 15 per cent. solution. In *Hygienische Rundschau* 1909, 12, Haserodt describes a fairly simple and practical way of demonstrating scanty tubercle bacilli in sputum, which is a combination of the antiformin method of Uhlenhuth and the ligroin method of Lange and Nitsche. The sputum is thoroughly mixed with four to five times its volume of a 5 per cent. solution of antiformin, and allowed to stand for 24 hours at room temperature, or for 10 hours at 37° C. Then, after shaking up, 2 c.cm. of ligroin are added to the mixture, and this is stirred in a suitable glass vessel until a thick emulsion forms. After 10 minutes in a water bath at about 60° C, the ligroin separates off, being frothy at the top and in a clear layer below this. Through these layers is now passed a platinum loop, and a number of loopfuls taken from the boundary layer of the fluids. The slide, or cover glass, must be previously heated, and passing it three times through a flame suffices for fixation. In this way, it is claimed, good results can be obtained without a centrifuge.

---

Thornton, in the *Journal of the American Medical Association*, mentions the case of a virgin 32 years of age, who after menstruating regularly from the age of 13, suddenly became Amenorrhœic after a severe Attack of Mumps. From the onset of this attack the menstrual flux has been replaced by mammary congestion, accompanied by a discharge from the nipples of a mixture of blood and serum, sufficiently abundant in quantity to necessitate the use of absorbent cotton-wool bandages. Internal examination showed that the vagina was rudimentary, and that the uterus and its appendages were atrophied.

---

An interesting case of Premenstrual Nervous Asthma of thyroid origin is reported by Gennari in *Il Morgagni*. A young girl of 18, without any other physical defect than a marked hypertrophy of the thyroid gland, had generally, two or three days before her menstrual periods, attacks of asthma of sufficient intensity to necessitate medical interference. At the same time the thyroid body was vascular and tumid, and sibilant râles could be heard all over the lungs. Drugs had but little effect on the condition, which, however, passed away immediately the catamenia was established. The use of thyroid extract brought about the reduction in size of the gland to the normal, the asthma disappearing also. The author considers that this latter condition was the result of a reflex vaso-motor neurosis upon which the anomalous thyroid body had a great influence.

---

Falbing, in the *Hospitalstidende*, reports a case of Post-mortem Cæsarian Section with a living child. The operation was carried out one minute after the death of the mother from tuberculous meningitis. It is interesting to note that despite the long death agony of the mother, which lasted five or six hours, and was attended by marked circulatory disturbance, but little harm was done to the child, a female weighing 5½ lb. Birth occurred about three weeks before term. The infant has since then developed very satisfactorily.

Svehlen, in the *Archives für Hygiene*, reports the results of experiments he has made with regard to the Sterilisation of Dress Fabrics, by ironing them with a hot iron at a temperature of 196-312° C. The articles used in the experiments comprised pieces of linen, cotton and wool fabrics. These were first contaminated in various ways, such as by steeping them in river-water, using them as dusters, placing them in the beds of children suffering from infectious diseases, or by directly infecting them with pure cultures of bacillus typhosus, b. Diphtheriæ, streptococcus and staphylococcus. The materials were then damped and ironed, after which they were rubbed on culture media. It was thereby found that a single ironing with a well-heated iron is sufficient to thoroughly sterilise fine materials, such as those from which pocket-handkerchiefs are made. Thicker fabrics require several ironings on both sides before sterilisation is complete. The author therefore recommends ironing as a means of ensuring asepsis in the ordinary course of private practice, and more especially as a useful method of disinfecting the clothes of the practitioner attending cases of infectious disease.

---

Contrary to the findings of recent observers with regard to the Hepatic and Muscular Content in Glycogen during Pregnancy, Dele Chaie (*Archiv. ital. di Ginecolog.*) has observed a diminution of reserve glycogen in both liver and muscles. The total content of both liver and muscles in glycogen is, moreover, less than that contained in the liver alone of the non-pregnant female. The author attributes the erroneous conclusions of his predecessors to the fact that they experimented on herbivorous animals, whereas his own conclusions were based on results obtained from observations on the bitch. The diminution of reserve glycogen in the organism during pregnancy is not due to a slowing of the hepatic activity, but to a larger consumption of carbohydrates by the organism, as much for the needs of the fœtus as in consequence of increased oxidising processes.

---

As a Practical Variant on the usual method of performing Calmette's Ophthalmo-reaction, Wolff-Eisner, in the *Münch. Med. Woch.*, proposes to substitute a mixture of tuberculin in vaseline for the aqueous solution. This should be made up to a strength of 1 to 2 per cent. tuberculin. It keeps sterile indefinitely, and is, therefore, of great use in private practice. All accidental infections, which are the sole cause of the complications which occasionally follow the ophthalmo-reaction, are thereby avoided. The method of application of the ointment is very simple. By means of a glass rod, previously sterilised, a small quantity of the vaseline mixture about the size of a pea is introduced into the conjunctival sac, the lower eyelid at same time being kept away from the eye to allow of the ointment becoming spread over a large surface of the conjunctiva.

